# Effect of Action Observation Therapy in the Rehabilitation of Neurologic and Musculoskeletal Conditions: A Systematic Review

**DOI:** 10.1016/j.arrct.2021.100106

**Published:** 2021-01-27

**Authors:** Deirdre Ryan, Brona Fullen, Ebonie Rio, Ricardo Segurado, Diarmiad Stokes, Cliona O’Sullivan

**Affiliations:** a UCD School of Public Health, Physiotherapy, and Sports Science, Dublin, Ireland; b School of Allied Health, La Trobe University Melbourne, Melbourne, Victoria, Australia; c University College Dublin, Dublin, Ireland

**Keywords:** Neuronal plasticity, Rehabilitation, Systematic review, ADL, activities of daily living, AHA, Assisting Hand Assessment, AOT, action observation therapy, BBS, Berg Balance Scale, BBT, Box and Block Test, FOG, freezing of gait, ICF, International Classification of Functioning Disability, and Health, MAS, Modified Ashworth Scale, MCID, minimum clinically important difference, MD, mean difference, MDC, minimal detectable change, MI, motor imagery, MNS, mirror neuron system, MUUL, Melbourne Assessment of Unilateral Upper Limb Function, OM, outcome measures, PDQ-39, 39-item Parkinson Disease Questionnaire, RoB, risk of bias, ROM, range of motion, SF-36, Short Form-36 Health Survey, 10MWT, 10-m walk test, TUG, Timed Up and Go, UPDRS, Unified Parkinson Disease Rating Scale, VAS, Visual Analog Scale, WOMAC, Western Ontario McMaster Universities Osteoarthritis Index

## Abstract

**Objective:**

To investigate the effect of action observation therapy (AOT) in the rehabilitation of neurologic and musculoskeletal conditions.

**Data Sources:**

Searches were completed until July 2020 from the electronic databases Allied and Complementary Medicine Database (via OVID SP), Cumulative Index to Nursing and Allied Health Literature, Cochrane Library, EMBASE, MEDLINE, and the Physiotherapy Evidence Database.

**Study Selection:**

Randomized controlled trials comparing AOT with standard care were assessed. Musculoskeletal (amputee, orthopedic) and neurologic (dementia, cerebral palsy, multiple sclerosis, Parkinson disease, stroke) conditions were included. There were no age limitations. Articles had to be available in English.

**Data Extraction:**

Two reviewers independently screened titles, abstracts and full extracts of studies for eligibility and assessed the risk of bias of each study using the Cochrane Risk of Bias Tool. Data extraction included participant characteristics and intervention duration, frequency, and type.

**Results:**

The effect of AOT in different outcome measures (OMs) was referenced in terms of body structures and functions, activities and participation, and environmental factors as outlined by the International Classification of Functioning, Disability, and Health (ICF). Of the 3448 articles identified, 36 articles with 1405 patients met the inclusion criteria. Seven of the 11 meta-analyses revealed a significant effect of intervention, with results presented using the mean difference and 95% CI. A best evidence synthesis was used across all OMs. Strong evidence supports the use of AOT in the rehabilitation of individuals with stroke and Parkinson disease; moderate evidence supports AOT in the rehabilitation of populations with orthopedic and multiple sclerosis diagnoses. However, moderate evidence is provided for and against the effect of AOT in persons with Parkinson disease and cerebral palsy.

**Conclusions:**

This review suggests that AOT is advantageous in the rehabilitation of certain conditions in improving ICF domains. No conclusions can be drawn regarding treatment parameters because of the heterogeneity of the intervention. AOT has been considerably less explored in musculoskeletal conditions.

In recent years, rehabilitation interventions have evolved to reflect new understandings of neuroscience.^1^ Neuroplasticity refers to the ability of the nervous system to adapt in response to environmental or physiological changes and experiences.^2^ These changes can present within the structure, function, or organization of the nervous system and may occur centrally or peripherally. Cortical reorganization can result from structural lesions within the brain and from periods of disuse or pain.^3^^,^^4^ This ability to reorganize can be considered adaptive or maladaptive depending on whether it is associated with an increase or decrease in function. Restoration of maladaptive neuroplasticity may need to be actively targeted in rehabilitation programs to have the greatest chance of restoring functional abilities.^5^ Neurophysiological findings in recent times have led to the emergence of novel treatment strategies that address cortical reorganization. The discovery of the mirror neuron system (MNS) is one such advancement,^6^ which has led to the development of action observation therapy (AOT).

The MNS refers to a series of neurons distributed throughout the brain. This particular set of neurons activate both when one observes an action being performed or when one physically performs the action themselves.^6^ The core locations of the MNS lie within the inferior frontal gyrus, dorsal premotor and inferior parietal cortex, supplementary motor area, and the supplementary temporal gyrus.^6^ The MNS was first discovered in macaque monkeys when they observed another monkey or an experimenter perform an action.^7^ This prompted the exploration for a similar system within humans, which was subsequently discovered in the early 1990s.^8^ The presence of this cortical network is supported by brain imaging, electroencephalography, magnetoencephalography, and transcranial magnetic stimulation studies.^9^

Over the past 2 decades, AOT has become a well-substantiated therapeutic treatment in the field of neurorehabilitation but has been minimally investigated in patients with musculoskeletal conditions.^10^ AOT, which is the systematic observation of movements, facilitates engagement of the motor system as attention and is directed toward the central mechanisms that influence movement quality, promoting the reorganization of cortical changes and the restoration of cognitive references.^1^ Thus, AOT can lead to motor learning and the building or rebuilding of a motor memory via the MNS. AOT can be performed in isolation (observing the movement only) but more commonly is followed by the physical practice of the observed movements. Individuals with limited motor ability can participate in AOT, and so adaptive plasticity can still be promoted despite physical limitations.^10^ Additionally, AOT can be performed independently by patients and so maximizes the Physiotherapists time.

Despite the widespread use of AOT across a range of conditions and environments, a consensus has not yet been formulated on the optimal parameters in the implementation of this technique. The aims of this systematic review are therefore to (1) systematically review the effectiveness for AOT in improving impairment and functional outcomes in patients with neurologic and musculoskeletal conditions and (2) establish whether optimal parameters for the administration of AOT exist.

## Methods

The protocol of this review was registered and published at PROSPERO, https://www.crd.york.ac.uk/prospero/registration number CRD42018116029.

### Search strategy

A literature search was performed with the assistance of a medical librarian using the following electronic databases: Allied and Complementary Medicine Database (via OVID SP), Cumulative Index to Nursing and Allied Health Literature, Cochrane Library, EMBASE, MEDLINE, and the Physiotherapy Evidence Database. The search strategy was limited from 2008 to July 2020 and the English language only. Previously identified search terms were used; additionally each database was analyzed for predefined Medical Subject Headings of the National Library of Medicine terms. To ensure relevancy, a proximity search of 5 words was used. The following are examples of the search terms used: “action observation,” “visual feedback,” “action simulation,” “motor simulation,” and “mirror neuron∗.”

### Study identification

**Fig 1:**
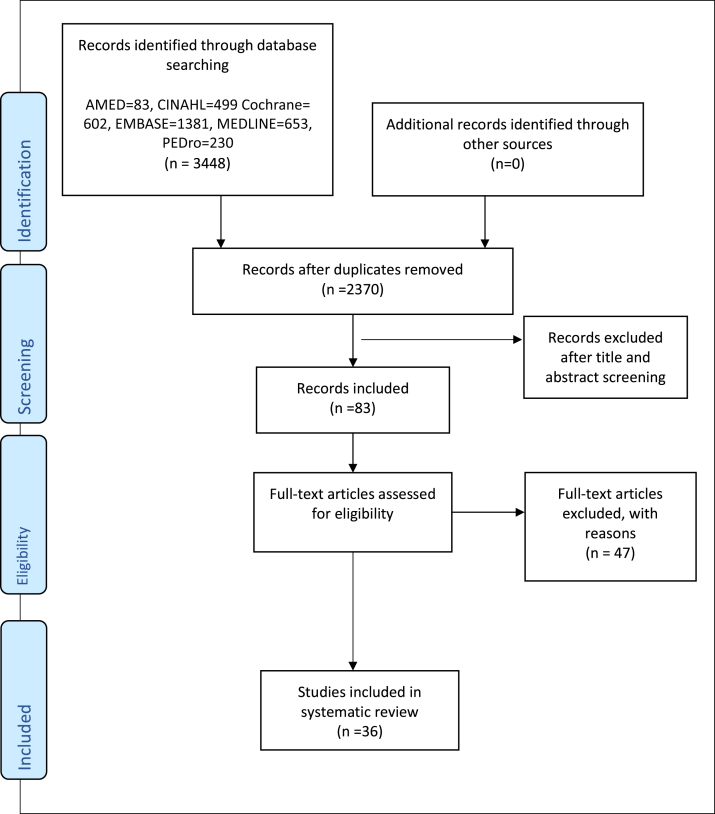
PRISMA flowchart. Abbreviations: AMED, Allied and Complementary Medicine Database; CINAHL, Cumulative Index to Nursing and Allied Health; PEDro, Physiotherapy Evidence Database; PRISMA, Preferred Reporting Items for Systematic Reviews and Meta-Analyses.

Articles retrieved in the initial search strategy were imported into EndNote, the reference management software. After the cross-referencing and removal of duplicates, the remaining articles were screened by title and abstract by 2 independent researchers. The references were selected following the inclusion and exclusion criteria (box 1). Eligible articles were sourced in full text and independently read by the same 2 researchers. The final number of articles that fulfilled the criteria was selected through discussion (fig 1). No disagreements arose in the selection process, and so no third party was consulted. Data detailing participant characteristics along with the duration, frequency, and type of intervention were extracted from the included studies.

**Box 1:** Inclusion criteria

Inclusion Criteria	Exclusion Criteria
• Randomized controlled trials Participants • All ages • All genders • All musculoskeletal & neurological conditions Intervention • A course of AOT (watching a video or person) Outcome measures • Body Structure and Function • Activity and Participation • Environmental Factors Comparisons • Control group	Intervention • Other forms of therapy that activate the MNS (virtual reality, mirror therapy) • Studies where brain imaging was the only OM assessed (to ensure clinical applicability)

### Risk of bias

**Fig 2:**
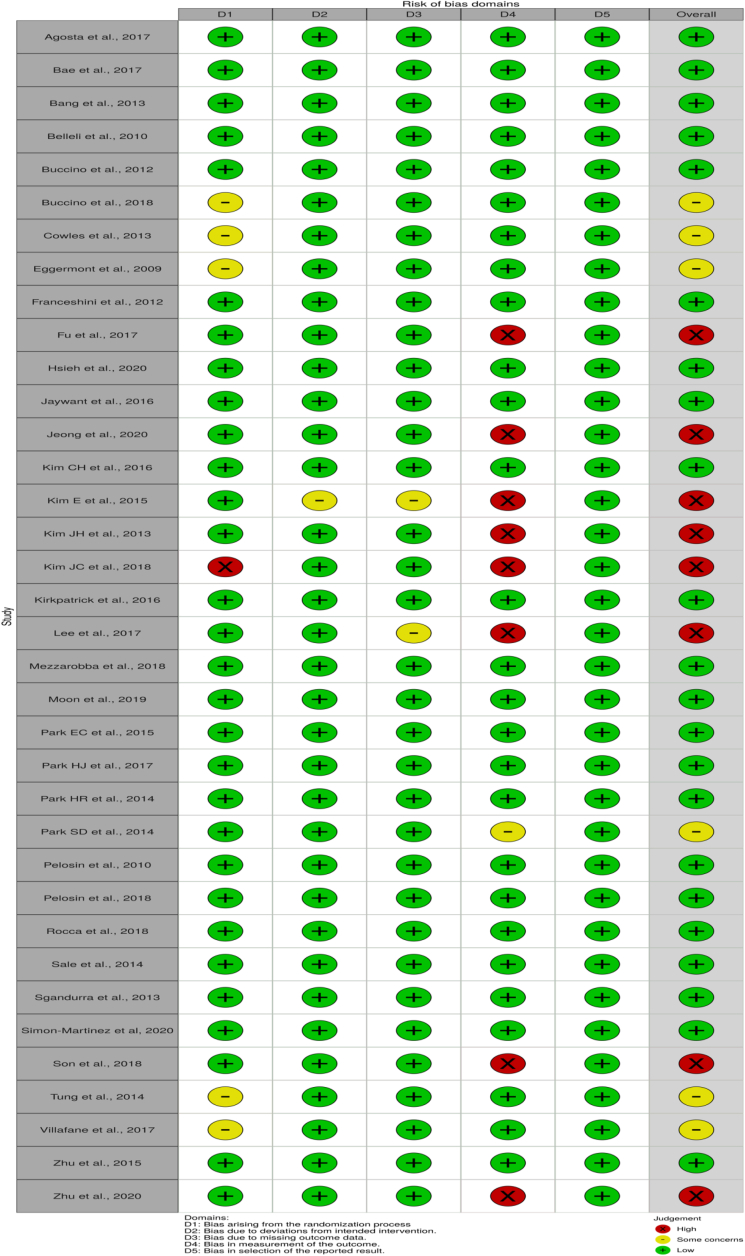
Risk of bias.

The Cochrane Risk of Bias (RoB) 2.0 tool (table 8.5a in the Cochrane Handbook for Systematic Reviews of interventions)^11^ was used by the 2 independent researchers to assess the RoB of each study. Any disagreement encountered was resolved through discussion. The RoB was classified as high, low, or some concerns in accordance with the criteria. The domains assessed are outlined in fig 2. Results are displayed using the robvis tool.^48^

### Data synthesis

As a particular strength of the International Classification of Functioning, Disability, and Health (ICF) is its focus on the functioning abilities of the individuals, recognizing the interaction between an individual’s health condition, personal factors, and environmental factors, the ICF will be referenced as a framework to articulate the findings of this review.^49^ A best evidence synthesis was used across the outcome measures (OMs). This qualitative analysis was performed based on a modified version of the 5 levels of evidence as outlined by van Tulder (box 2).^50^ For this synthesis, studies with a low RoB were considered high quality, while studies with some concerns or a high RoB were considered low-quality studies. Where studies provided sufficient homogeneity, a meta-analysis was performed in RevMan 5.3 using a random effects model. Treatment effect was calculated using mean difference (MD) with 95% CIs. The MDs were calculated using the reported pre- and post means, selecting the most comparable time point in cases where there were multiple follow-up time points. SDs for the mean change were calculated using the following formula:

$$s_{post-pre}=\sqrt{s_{pre}^{2}+s_{post}^{2}-2rs_{pre}s_{post}}$$

where *s* is the reported SD and *r* is the Pearson correlation coefficient between pre- and postscores. As these correlations are very rarely reported, where they were not provided, a conservative estimate of *r*=0.5 was used. Forest plots were created using this information, and the *I*^2^ statistic was used to assess heterogeneity. Treatment effect was compared with the minimal detectable change (MDC) or the minimum clinically important difference (MCID) values where these values are available.

**Box 2:** Modified version of van Tulder levels of evidence

1. Strong evidence(Level 1): consistent findings in ≥2 studies with a low risk of bias (≥75% of the studies reported consistent findings).
2. Moderate evidence(Level 2): consistent findings in multiple studies with some concerns or high risk of bias or one study with a low risk of bias (≥75% of the studies reported consistent findings).
3. Limited evidence(Level 3): consistent findings in ≥1 study with some concerns or high risk of bias (≥75% of the studies reported consistent findings).
4. Conflicting evidence(Level 4): inconsistent findings in multiple studies (<75% of the studies reported consistent findings).
5. No evidence(Level 5): no studies could be found.

## Results

### Characteristics of included studies

Four studies evaluated musculoskeletal conditions: amputees (n=1) and orthopedic hip or knee replacement surgery (n=3). Thirty-two studies evaluated neurologic conditions: cerebral palsy (n=6), dementia (n=1), multiple sclerosis (n=1), Parkinson disease (n=5), or stroke (n=19).

Thirty-five studies were parallel randomized controlled trials,^12^, ^13^, ^14^, ^15^, ^16^, ^17^, ^18^, ^19^, ^20^, ^21^, ^22^, ^23^, ^24^, ^25^, ^26^, ^27^^,^^29^, ^30^, ^31^, ^32^, ^33^, ^34^, ^35^, ^36^, ^37^, ^38^, ^39^, ^40^, ^41^, ^42^, ^43^, ^44^, ^45^, ^46^, ^47^ and 1 study^28^ was a crossover randomized controlled trial. The studies included a total of 1405 participants, with sample sizes ranging from 15-102 participants. The age range of participants was expansive, spanning 3-91 years. The duration of intervention varied from 8 days^23^ to 12 weeks,^27^ with 4 weeks being the most common duration as preferred in 15 studies (table 1). The frequency varied from twice a week^31^ to 7 days a week,^18^^,^^23^^,^^39^^,^^41^^,^^42^^,^^44^ with 5 days being the most common frequency. Equally, varying time periods were seen across all studies ranging from 10-minute^35^ to 60-minute sessions,^12^^,^^22^^,^^31^^,^^37^^,^^41^^,^^42^ with 30-minute sessions the most common duration as selected in 15 studies. Six of the studies had AOT twice a day.^18^^,^^20^^,^^28^^,^^41^^,^^42^^,^^45^ Twelve studies completed a further follow-up after the posttreatment assessments, ranging from 1-6 months.^12^^,^^17^^,^^20^^,^^22^^,^^29^^,^^31^^,^^37^^,^^38^^,^^40^, ^41^, ^42^^,^^47^

**Table 1:** Study characteristics

Study	Domain	Participant Details	Therapy	Time & Frequency	Outcome Measures	Results
Tung et al^44^	Amputee	I: n=11 (11M) Mean age ± SD: 26.7±5.6 y C: n=9 (9M) Mean age ± SD: 26.7±5.6 y	I: Observed 7 lower limb movements while simultaneously attempting to copy the movements with their phantom limbs. C: Closed their eyes and attempted to move their phantom limbs while visualizing each of the movements as prompted by the investigator. Adjunct: Continue normal rehabilitation and medication.	20 min Daily 4 wk	Assessed prior to daily treatment (i) VAS 0-100 (ii) SF-MPQ (iii) No. and duration of PLP episodes over the past 24 h (iv) Changes in analgesic medications	(i) SD in I group (*P*<.05) Between-group score difference was 4 mm for the right leg and 18 mm for the left leg (ii) SD in I group (*P*<.05) Between-group score difference was 3.8 for the right leg and 4 for the left leg (iii) NS (iii)) NS
Belleli et al^15^	Orthopedic	I: n=30 (21F, 9M) Mean age ± SD: 71.9±8.4 y C: n=30 (16F, 14M) Mean age ± SD: 71.9±6.9 y	I: Observed 3 short movies showing an actor perform daily actions using the leg or trunk. Each movie included 4 different 2-min actions. After observations, patients executed the observed actions to the best of their ability. C: Observed video clips with no motor content and executed the same actions as I group afterward. Adjunct: Conventional PT 1h/d, 6d/wk for 3 wk.	24 min 6 d/wk for 3 wk	Assessed at baseline and post intervention; walking aids assessed weekly (i) FIM total score (ii) FIM motor and locomotion subscore (iii) Tinetti scale score (iv) Type/no. of walking aids.	(i) SD between groups (*P*<.05) Between-group MD: 6.4 (95% CI, .99-11.81) (ii) SD between groups (*P*=.002, *P*=.001, respectively) Between-group MD: 4.4 (95% CI, 2.06-6.74) (iii) SD between groups (*P*=.04) Between-group MD: 2.2 (−1.33 to 5.73) (iv) Use of a single crutch was also significantly different between groups (*P*=.01) with a 23.4% difference of 1 crutch use between groups
Park et al^35^	Orthopedic	I: n=9 Mean age: 72.67 y C: n=9 Mean age: 70.56 y	I: 8 tasks were presented via video (2-3min for each). Observed the tasks with no physical practice during the first wk, then performed the first 4 tasks 3 times during the following wk and the remaining 4 tasks during the third wk. C: Received PT for 30 min. Adjunct: None specified.	40 min 3 times/wk for 3 wk	Assessed at baseline and post intervention (i) WOMAC (including pain, stiffness, function) (II) TUG	(i) SD between groups in favor of the I group (*P*<.001). Group MD for pain: −4.32 (95% CI, −7.32 to −1.32); for function: −13.32 (95% CI, −18.97 to −7.67); and for stiffness: −1.89 (95% CI, −3.14 to −0.64) (ii) NS
Villafañe et al^45^	Orthopedic	I: n=14 (7F,7M) Mean age ± SD: 70.4±7.5 y C: n=17 (14F, 3M) Mean age ± SD: 70.1±7.7 y	I: Watched a video (13.38min) of exercises prior to independently physically performing the exercises. C: Watched a video of nature scenes and performed the same exercises as the I group. Adjunct: continuous passive motion applied twice/d for 20 min after treatment.	30-45 min Twice daily 5 d/wk for 2 wk	Assessed at baseline and post intervention (i) VAS (ii) Active and passive ROM of the knee (iii) Barthel Index (iv) SF-36 (v) Tinetti scale (vi) Lequesne index measures	(i) S effect for time in I group (*P*<.001). Small between-group effect size (*d*=0.1), mean difference: 2.5 (95% CI, −15.5 to 20.2) (ii) S effect for time in I group for active and passive ROM (*P*<.001) with a between-group MD of 15.6° (95% CI, 5.3-24.8) for active flexion and 3.4° (95% CI, 1.1-5.6) for active extension. Large between-group effect sizes (*d*>1.3). Moderate-large effect size was seen for passive ROM (*d*=0.3-0.7) (iii) S effect for time in I group (*P*<.001). Moderate between-group effect size (*d*=0.7) (iv) NS. SF-36 motor between-group MD: 5.8 (95% CI, −0.7 to 12.3). SF-36 mentality: 3.9 (95% CI, −4.5 to 12.2) (v) S effect for time in I group (*P*<.001). Large between-group effect size (*d*=1.2). Between-group MD: 2.9 (95% CI, 0.8-5.0) (vi) S effect for time in I group (*P*<.001). Large between-group effect size (*d*=0.9). Between-group MD: −3.4 (95% CI, −6.4 to −3.5)
Buccino et al^16^	Cerebral palsy	I: n=8 (4F, 4M) Mean age: 7y 6 mo C: n=7 (2F, 5M) Mean age: 8 y Participants had hemiplegia or diplegia	I: Watched 12-min videos of arms/hands performing 3-4 motor acts. Physical practice for 2 min after each motor segment. C: Videos (history, geography). No motor content. Physical practice after performing same exercises as I group. Adjunct: Children continued to follow their routine conventional rehabilitation program.	15-20 min 5 d/wk for 3 wk	Assessed twice (T1,2) at baseline (2wk apart) and no later than 2 d after the end of treatment (T3) (i) Melbourne Assessment Scale	(i) SD between groups in favor of the I group (*P*=.026). Between-group MD at T3: 12.679
Buccino et al^17^	Cerebral palsy	I: n=11 (6F, 5M) Age range: 5-11 y C: n=7 (3F, 4M) Age range: 5-11 y Participants had hemiplegia or tetraplegia	I: 15 video clips showing specific daily actions using arms/hands. Each action presented for 3 min in 3-4 motor segments. Execute movement after each motor segment for 2 min. C: Watched geography, history, and science video clips for 3 min. No motor content. After observing each segment, the same physical movements were executed. Adjunct: Continued to follow their routine conventional rehabilitation program.	30 min 5 d/wk for 3 wk	Assessed at baseline (T1), post intervention (T2), and 2-mo follow-up (T3) (i) MUUL (ii) AHA	At T3 treated children maintained and even improved their functional gain at follow-up. (i) SD between groups in favor of the I group (*P*<.001). Between-group MD at T3: 5.77 (95% CI, −12.3 to 23.84) (ii) SD between groups in favor of the I group (*P*<.001) Between-group MD at T3: 4.73 (95% CI, −4.1 to 13.53)
Jeong & Lee^24^	Cerebral palsy	I: n=9 (6F, 3M) Mean age: 7.44±1.88 y C: n=9 (4F, 5M) Mean age: 6.90±1.79 y Participants had diplegia	I: Watched videos of movements for 15 min, followed by 5-min practice C: General PT given 5 times/wk, for 30 min for 6 wk including transitioning of positions	30 min 3 d/wk for 6 wk	Assessed pre- and post intervention (i) Ankle stiffness (ii) Modified Tardieu Scale (iii) Gross Motor Function Measure-88 (scales A-E) (iv) Pediatric Arm Reach Test	(i) NS (ii) NS (iii) SD between groups for GMFM-E; between-group MD: 5.38 (95% CI, 2.5-8.26) (iv) SD between-group (cm) MD: lateral right 1.88 (95% CI, 0.29-3.47) lateral left 2.66 (95% CI, 1.17-6.79) frontal right 2.58 (95% CI, 0.23-4.93) frontal left 2.21 (95% CI, 0.35-4.07)
Kirkpatrick et al^29^	Cerebral palsy	I: n=35 (18F, 19M) Mean age: 5 y 2 mo C: n=35 (13F, 21M) Mean age: 5 y 4 mo Participants had hemiplegia	I: Watched parent perform movement prior to attempting the same movement. Parent sat on the side of less affected hand. Received around 12 tailored activities. C: Control group played independently (with parental supervision). Adjunct: Diary given to record session details and reward stickers for the children. Families telephoned fortnightly for support.	15 min 5 d/wk for 12 wk	Assessed at baseline (T0), 3 mo, and 6 mo (i) AHA (ii) Melbourne Assessment 2 (iii) ABILHAND-Kids	(i) NS (ii) NS (iii) NS
Sgandurra et al^41^	Cerebral palsy	I: n=12 (4F, 8M) Mean age ± SD: 9.48±2.12 y C: n=12 (4F, 8M) Mean age ± SD: 9.94±2.77 y Participants had hemiplegia	I: Observed video of goal-directed actions (3min), then performed physical practice for 3 min. Same video sequence played twice. Every day, 3 different goal directed actions of increasing complexity were observed. Therapist sat on affected side to prompt attention during task. C: Watched computer games, then verbally instructed to perform the same actions in the same order as the experimental group. Adjunct: None specified.	60 min 15 consecutive d	Assessed at baseline (T0), 1 wk (T1), 8 wk (T2), and 24 wk after the end of training (T3) (i) AHA (ii) MUUL (iii) ABILHAND-Kids	(i) At T3 between-group MD: 1 (95% CI, −0.37 to 2.37) (ii) NS (iii) NS
Simon-Martinez et al^42^	Cerebral palsy	I: n=22 (7F, 15M) Mean age ± SD: 9 y 6 mo±1 y 11 mo C: n=22 (10F, 12M) Mean age ± SD: 9 y 6 mo±1 y 10 mo Participants had hemiplegia	I: Video watched for 3 min, children executed the observed task for 3 min. This was done for 3 activities and repeated twice for each activity; 18 min total. C: Watched video games of free human movement, then executed the same movements in the same order as the I group.	60 min 9/11 d 15 (either 1-2 sessions/d)	Assessed at baseline 3-4 mo before intervention (T0), within 4 d before intervention (T1), within 4 d after intervention (T2), and 6 mo after intervention (T3) (i) AHA (ii) MAS (ii) Muscle strength (8-point ordinal scale of the Medical Research Council) (iii) Grip strength using the hand dynamometer (iv) Melbourne Assessment 2 (v) Modified version of the Jebsen-Taylor Hand Function Test (vi) Tyneside Pegboard Test (vii) ABILHAND-Kids (viii) Children’s Hand-use Experience Questionnaire	(i) NS (ii) NS (iii) NS (iv) NS (v) NS (vi) NS (vii) NS (viii) NS
Eggermont et al^19^	Dementia	I: n=19 (18F, 1M) Mean age ± SD: 84.8±5.2 y C: n=25 (24F, 1M) Mean age ± SD: 86.4±5.2 y	I: Participants in groups of 4 watched videos of hands of a person performing creative activities. C: Participants watched 10 videos from a documentary on Dutch provinces. Adjunct: None specified.	30 min 5 d/wk for 6 wk	Assessed at baseline, wk 6, and wk 12 (i) Memory assessed with face recognition, picture recognition, and 8 words test (ii) Executive function assessed with 2 tests: the digit span (iii) Category fluency	(i) NS. An interaction effect shown for face recognition in I group (*P*=.006). (ii) NS between groups. The digit span showed an S interaction effect. (iii) NS
Rocca et al^39^	MS	I: n=20 (11F, 9M) Median age: 50.4 y C: n=21 (15F, 6M) Medial age: 51.5 y	I: Watched 3 videos (5min each), then execution of right-hand daily life activities for 5 min. 10-min right upper limb passive mobilization prior to viewing videos. C: Watched videos of inanimate landscape videos. Execution of the same upper movements as I group. Adjunct: Patients with MS underwent a 40-minute daily standard rehabilitation session.	40 min daily for 2 wk	Assessed at baseline and after 2 wk (±1d) (i) Hand muscle strength (Jamar and pinch dynamometers) (ii) Manual dexterity (9-hole peg test) and 30-s finger tapping frequency. (iii) Cognitive function: Paced Auditory Serial Addition Test	(i) SD between groups for right Jamar (*P*=.04). Between-group MD of 1kg. (ii) NS (iii) NS
Agosta et al^12^	PD	25 consecutive right-handed patients with PD 2 groups I: n=12 10M, 2F Mean age ± SD: 69±8 y Landscape: n=13 (8M, 5F) Mean age ± SD: 64±7 y. 19 age- and sex-matched righted controls without PD Idiopathic PD, level 1-3 on the Hoehn and Yahr scale, duration at least 5 y	I: Video clips showing strategies helpful in circumventing FOG episodes (6min) presented twice. After each video clip, physical practice performed for 12 min repetitively and accurately at the beat of the auditory cueing. The complexity of the actions progressively increased. C: Watched videos of static landscapes. Physical practice of exercises after, which matched I protocol. Adjunct: Participants were allowed to continue their ordinary motor activities; asked not to practice or undertake any specific PT and no change in medication permitted.	60 min 3/wk for 4 wk	Assessed at baseline, wk 4, and wk 8 (i) UPDRS (ii) PDQ-30 (iii) FOGQ (iv) Hoehn and Yahr scale (v) BBS (vi) 10MWT	(i) SD. Between-group MD at W8: −2.9 (95% CI, −10.46 to 4.66) (ii) SD in I group only at wk 8 (*P*<.001). Between-group MD at W8: −4.2(95% CI, −12.72 to 4.32) (iii) NS (iv) NS (v) Between-group MD at W8: 0.30 (95% CI, −2.62 to 3.22) (vi) NS
Jaywant et al^23^	PD	I: n=13 (7F, 6M) Mean age ± SD: 63.7±6.2 y C: n=10 (6F, 4M) Mean age ± SD: 65.8±8.7 y Idiopathic PD, level 1-3 on the Hoehn and Yahr scale	I: Watched videos of actors walking in a hallway. Participants judged via keyboard press whether the walking appeared healthy or resembled a PD-like gait pattern. Feedback (correct or incorrect) was presented after each trial. The same videos appeared daily in a randomized order. C: Viewed videos of landscapes with moving water. Participants took home a laptop computer. They judged the videos via keyboard press. Feedback (correct or incorrect) was presented after each trial. The same videos appeared daily in a randomized order. Adjunct: None outlined.	Daily for 8 d	Assessed at baseline and 7 d after completion of the home-based training (i) Spatiotemporal walking variables were assessed using accelerometers in the laboratory; daily activity, walking speed, stride length, stride frequency, leg swing time, and gait asymmetry. (ii) PDQ-39	(i) NS (ii) SD between groups (*P*<.01). Between-group MD: −8.56 (95% CI, −26.313 to 9.19)
Mezzarobba et al^31^	PD	I: n=12 (5F, 7M) Mean age ± SD: 74.67±5.93 y C: n=10 (3F, 7M) Mean age ± SD: 72±5.87 y (3F, 7M) Idiopathic PD, level 1-3 on the Hoehn and Yahr scale	I: 8 videos (each 1.5min) showing 8 motor gestures were presented. Physical practice performed of same movements for 1.5 min. Each video was composed of images and sounds. All videos presented in each session from simple to complex. Each video repeated twice. C: Same 8 motor gestures performed in the same order for the same amount of time. Patients asked to practice via visual or auditory cues. Physiotherapist corrected and assisted in facilitating correct motor patterns. Adjunct: Instructed not to practice further rehabilitation/PT during the duration of the study.	60 mins 2/wk for 8 wk	Assessed at baseline, post intervention, 1-mo follow-up, and 3-mo follow-up (i) NFOGQ (ii) UPDRS (iii) PDQ-39 (iv) TUG (v) 6MWT (vi) BBS (vii) Modified Parkinson Activity Scale	(Unable to determine mean [95% CI]) (i) SD between groups in favor of I group at 1st and 2nd follow-up (*P*<.001) (ii) SD between groups in favor of I group at 1st and 2nd follow-up (*P*<.05) (iii) SD between groups in favor of I group at 1st and 2nd follow-up (*P*<.01) (iv) NS (v) SD between groups in favor of I group at 2nd follow-up (*P*<.05) (vi) SD between groups in favor of I group at 1st follow-up (*P*<.05, NS at 2nd follow-up) (vii) NS
Pelosin et al^37^	PD	I: n= 9 Mean age ± SD: 68.8±4.1 y C: n=9 Mean age ± SD: 70.2±6.8 y Idiopathic PD, <3 on the Hoehn and Yahr scale	I: Watch 6 videos (6min) of strategies for circumventing FOG. 2 different videos presented twice and complexity of actions increased over the sessions. Under the supervision of a Physiotherapist. C: Landscape videos combined with the same physical practice under the supervision of a Physiotherapist. Adjunct: None specified.	60 min/wk 3/wk for 4 wk	Assessed at pre-, post intervention, follow-up (wk 1 follow-up, wk 2 follow-up, wk 3 follow-up, and wk 4 follow-up) (i) FOGQ (ii) No. of FOG episodes (iii) TUG (iv) 10MWT (v) Tinetti scale part 1 (vi) PDQ-39	(i) SD in favor of I group post intervention (*P*<.05). Between-group MD: −2.4 (95% CI, −4.2 to −0.6) (ii) No. of FOG episodes was SD at FW follow-up in I group (*P*<.05). (iii) NS (iv) NS (v) NS (vi) NS
Pelosin et al^38^	PD	64 patients with PD I: n=33 (17F, 16M) Mean age ± SD: 70.4±4.5 y C: n=31 (16F, 15M) Mean age ± SD: 72.8±3.1 y Idiopathic PD, level 2-3 on the Hoehn and Yahr scale	I: Group-based training, watched 6 videos (6min each) of strategies for circumventing FOG. 2 different videos presented twice and complexity of actions increased over the sessions. Physical practice of same movements performed after under the supervision of a Physiotherapist. C: Group-based training. Watched 6 videos of static landscapes. Performed the same actions in the same order as the I group under the supervision of a Physiotherapist. Adjunct: None specified.	45 min 2/wk for 5 wk	Assessed at baseline, within 1 wk post intervention, and 4-wk follow-up (i) UPDRS (ii) FOGQ (iii) TUG (iv) 10MWT (v) BBS	(i) NS (ii) NS post intervention. Meta-analysis performed post intervention. Between-group MD post intervention: −0.5 (95% CI, −3.17 to 2.17) SD between-group MD at 4-wk follow-up (*P*<.001): −2.3 (95% CI, −5.06 to 0.46) (iii) SD at 4-wk follow-up in I group only (*P*<.001) Between-group MD post intervention: 0.00 (95% CI, −3.36 to 3.36) (iv) NS (v) SD at 4-wk follow-up in I group only (*P*<.001). Meta-analysis performed post intervention, MD: 0.9 (95% CI, −2.48 to 4.28)
Bae et al^13^	Stroke	I: n=9 (4F, 5M) Mean age ± SD: 49.50±10.60 y C: n=9 (5F, 4M) Mean age ± SD: 49.67±8.78 y Chronic stroke (6- 24mo since event), patients with hemiplegia	I: DASI group To provide motivational stimuli, the DASI group watched previous recordings of dorsiflexion of the contralateral ankle for 20 min and were instructed to imitate the movement. Participants performed movement during the ETFES application. C: A microstimulation device was used to apply FES in the control group for 20 min. The patient was instructed to perform dorsiflexion on FES application. The placement of the electrodes and the electrical current were identical in both groups. Adjunct: All participants received general PT for 30 min daily, 5 d/wk.	20 min 5 d/wk for 4 wk	Assessed at baseline, post intervention, 2-wk follow-up, and 4-wk follow-up (i) Movement-related cortical potential measured using the QEEG-8 at C3, Cz, and C4 (ii) H-reflex measured using Neuro-EMG-Micro. Active electrode: head of gastrocnemius, reference electrode Achilles tendon. (iii) EMG using a wireless BTS pocked (iv) BioRescue system used to measure balance	(i) SD in motor potential between groups in favor of the I group at C4. MD: 2.51 (95% CI, 1.10-3.92) (ii) H-reflex was S decreased in the C group after 4 wk. H-reflex was significantly reduced in the I group after 2nd and 4th wk of training. (iii) NS between groups (iv) SD between groups in dynamic balance in favor of the I group (*P*<.05). MD: 29.63 (95% CI, –0.64 to 54.90)
Bang et al^14^	Stroke	I: n=15 (6F, 9M) Mean age ± SD: 64.1±6.35 y C: n=15 (7F, 8M) Mean age ± SD: 58.9±7.03 y Chronic stroke (>6 mo since event), patients with hemiplegia, 1st stroke	I: Group watched a video showing treadmill training. Video divided into 3 phases. Each phase contained the walking actions of a healthy person and provided 3 different views. Video was shown at a normal speed for 3 min, half speed for second 3 min, and normal speed for last 3 min. After watching the video, the participants had time to organize their thoughts for 1 min after which they performed treadmill exercise for 30 min. C: Received treadmill training after watching a nature video. The group was provided with the same protocol as the I group. Adjunct: None specified.	40 min 5 d/wk for 4 wk	Assessed at baseline and post intervention (i) TUG (ii) 10MWT (iii) 6MWT (iv) Maximal flexion knee angle in the swing phase during walking measured using a camera system	Significant improvements in (i) SD between groups in favor of the I group (*P*=.018). Very large effect size (1.27). Between-group MD: −2.22 (95% CI, −3.50 to −0.94) (ii) SD between groups in favor of I group (*P*=.001). Medium effect size (0.57). Between-group MD (m/s): 0.20 (95% CI, −0.10 to 0.50) (iii) SD between groups in favor of I group (*P*=.001). Huge effect size (2.34). Between-group MD: 60.60 (95% CI, 48.43-72.77) (iv) SD between groups in favor of I group (*P*=.03). Small effect size (0.37). Between-group MD: 2.57 (95% CI, −2.37 to 7.51).
Cowles et al^18^	Stroke	I: n=15 (7F, 8M) Mean age ± SD: 78.8±8.1 y C: n=15 (5F, 9M) Mean age ± SD: 75.6±12.4 y Acute stroke, population with hemiplegia	I: Watched the therapist perform a functional task for 1-2 min and to think about copying in preparation for doing exactly the same movement in time with the therapist for 4-6 min. 8-min periods divided by 2-4 min of resting. Verbal correction was given. The therapist sat alongside the patient on the paretic side and used the upper limb that matched the participant’s paretic side. C: No videos or physical practice. Received conventional PT only. Adjunct: All participants received conventional PT as deemed appropriate by the clinical therapist.	2×30 min/d adjusted to 2×20 min/d 5/wk	Assessed at baseline and post intervention (i) Motricity Index (ii) Action Research Arm Test	(i) NS (ii) NS
Franceshini et al^20^	Stroke	I: n=53 (33F, 20M) Mean age ± SD: 67.0±12.4 y C: n=49 (21F, 28M) Mean age ± SD: 65.7±11.9 y Acute stroke, population with hemiplegia, 1st stroke	I: Patient watched video footage showing 20 different daily routine tasks carried out with the upper limb. Patient presented with 1 task/d, starting from the easiest and ending with the most complex. Each action consisted of 3 different motor sequences in order of ascending difficulty and lasting 3 min each. 2-min physical performances for the 3 sequences. Received verbal instruction by the OT, if needed the OT provided physical assistance. C: Participants shown 5 static images displaying objects. Participants then performed limb movements for 2 min, simulating those shown in the intervention group. Verbal instruction was provided by the OT, along with physical assistance if needed. Adjunct: All patients underwent 3 h of daily, including both dexterity and gait training.	2×15-min sessions 5/wk for 4 wk	Assessed at baseline (T0), post intervention (T1), and 4-5–mo follow-up (T2) (i) BBT (ii) Fugl-Meyer Assessment (iii) Frenchay Arm test (iv) Ashworth Scale elbow and wrist (v) FIM	(i) SD between groups in favor of I group from T0-T1 (*P*=.003) and T0-T2 (*P*=.010). Between-group MD at T1: 5.3 (95% CI, −1.24 to 11.84) (ii) NS (iii) NS (iv) NS (v) NS
Fu et al^21^	Stroke	I: n=28 (17F, 11M) Mean age ± SD: 62.04±9.93 y C: n=25 (14F, 11M) Mean age ± SD: 59.76±10.57 y Subacute-chronic (2-6 mo since event), population with hemiplegia, 1st stroke	I: 30 actions in the video, from simple to complex. Each action shown from 2 different angles for 50 s. Participants watched the videos for 10 min and then imitated the action for 10 min. C: Watched different geometric patterns and digit symbols then performed actions selected from the same videos as the intervention group. Adjunct: Patients in both groups were treated with drugs for medical purposes. Traditional PT was provided in both groups.	20-min sessions 6/week 8 weeks	Assessed baseline and post intervention (i) Fugl-Meyer Assessment (ii) Wolf Motor Function Test (iii) Modified Barthel Index (iv) Motor evoked potential	(i) SD between the groups, in favor of I groups (*P*<.05). Between-group MD: 5.38 (95% CI, −1.13 to 11.89) (ii) SD between groups in favor of I groups (*P*<.05). Between-group MD: 0.40(95% CI, −3.30 to 8.10). (iii) SD between groups in favor of I groups (*P*<.05). Between-group MD: 6.00 (95% CI, 0.14-11.86) (iv) NS
Hsieh et al^22^	Stroke	I: (AOT); n=7 (1F, 6M) Mean age ± SD: 52.77±11.25 y I: Mirror therapy; n=7 (1F, 6M) Mean age ± SD: 46.1±13.45 y Active control intervention (customary bilateral arm training): n=7 (1F, 6M) Mean age ± SD: 54.30±13.61 y Subacute-chronic (1-6 mo since event), population with hemiplegia	I: 3 phases each session. Phase 1 (10-15min) patients watched AROM exercises and simultaneously executed the movements. Phase 2 (15-20min) observed a reaching or object manipulation movement for 2 min, physically practiced for 3 min, repeated 3 times. Phase 3 (30min) 1 functional task, progressing from easy to complex. Observed movement for 2 min, practiced for 3 min. Repeated 3 times. Watched video in first person. Mirror therapy: AROM exercise (10-15min), reaching movement or object manipulation (15-20min) and functional task practice (30min). Instructed to watch mirror reflection of the movement performed by unaffected hand, encouraged to move affected hand as much as possible. Active control intervention-customary bilateral arm training: Received dose-matched bilateral arm training provided by an OT. No video or mirror input received. The same 3 categories of movement were received.	60 min 5/wk for 3 wk	All evaluations were performed at baseline (T0), immediately after treatment (T1), and at 3 mo after treatment (T2) (i) Fugl-Meyer Assessment (ii) BBT (iii) FIM (iv) Stroke Impact Scale	(i) NS. A total of 4, 1, and 5 patients achieved MCID in the I, mirror therapy, and active control groups, respectively. (ii) NS. A total of 4, 2, and 4 patients achieved MCID in the I, mirror therapy, and active control groups, respectively. (iii) 1 patient only achieved MCID, a patient in the I group. Between-group difference with active control group: 6.72 (95% CI, −6.87 to 20.31) (iv) NS. A total of 4, 4, and 4 patients achieved MCID in the I, mirror therapy, and active control groups, respectively.
Kim et al^25^	Stroke	I: n=11 (4F, 7M) Mean age ± SD: 60.77±7.03 y C: n=11 (5F, 6M) Mean age ± SD: 59.11±7.05 Subacute-chronic (1-6 mo since event), population with hemiplegia, 1st stroke	I: Watched videos (9min), divided into 3 phases, according to speed (normal, 50% normal and normal). Each video involved the same tasks and provided 3 views. After watching the videos, participants organized thoughts for 1 min and then performed physical practice for 30 min. C: Underwent task specific training without watching the video. The group practiced the same tasks as the I group during a 30-min period. Adjunct: All participants also received a conventional rehabilitation program that involved occupational (1h/d), physical (2h/d), and speech therapies as required. Duration and intensity was the same for both groups	40 min 5/week 4 weeks	All evaluations were performed before and immediately after treatment (i) Fugl-Meyer Assessment (ii) BBT (iii) Modified Barthel Index (iv) MAS	(i) SD between groups in favor of I groups (*P*<.05). Between-group MD: 4.23 (95% CI, 1.56-6.90) (ii) SD between groups in favor of I groups (*P*<.05). Between-group: 2.80 (95% CI, 0.85-4.75) (iii) SD between groups in favor of I groups (*P*<.05). Between-group MD: 7.44 (95% CI, 4.62-10.26) (iv) NS
Kim et al^26^	Stroke	12 participants I: Not provided C: Not provided Population with hemiplegia	I: The program was based on the study by Feys et al. The purposeful action observation program included activities of daily living. C: No details provided. Adjunct: No details provided.	30 min 5/wk for 6 wk	Assessed at baseline and post intervention (i) Wolf Motor Function Test	(i) SD between groups in favor of I groups (*P*<.05). Between-group MD: 0.60 (95% CI, −13.75 to 14.95)
Kim et al^27^	Stroke	I: n= 9 (2F, 7M) Mean age ± SD: 55.3±12.1 y Motor imagery group: n=9 (3F, 6M): Mean age ± SD: 54.8±8.8 y Physical training group: n=9 (2F, 7M) Mean age ± SD: 59.8±8.9 y Chronic, population with hemiplegia	I: Practiced additional 30 min to the physical training program. Training consisted of 4 stages (according to content and level of difficulty), each phase 1 wk long. Participants viewed a task video for 20 min followed by training with a therapist for 10 min based on the video. Video was produced separately for left and right hemiplegia. Motor imagery group: Practiced additional 30 min to the physical training program. Conducted for 20 min according to the motor imagery program played through a computer speaker and physical training for 10 min based on the training contents. The contents of the motor imagery program were identical to the contents in the action observation training program. Adjunct: All participants underwent neurodevelopmental therapy for 30 min, twice/d, 5 d/wk. Exercise program including transfers and walking patterns.	30 min 5/wk for 4 wk	Assessed at baseline and post intervention (i) TUG (ii) Functional reaching test (iii) Walking ability questionnaire (iv) Functional ambulation category (v) Spatiotemporal gait parameters were collected using a GAITRite system	(i) SD between I and physical training groups in favor of I groups (*P*<.05). NS between I group and motor imagery group. Between-group MD: −4.77 (95% CI, −16.14 to 6.60) (ii) NS (iii) NS (iv) NS (v) SD between I and physical training groups in favor of I group (*P*<.05) in gait speed, cadence, and single leg support on the affected side. NS between I group and motor imagery group.
Kim et al^28^	Stroke	I: n=11 (1F, 10M) Mean age ± SD: 57.08±7.29 y C: n=10 (1F, 9M) Mean age ± SD: 52.92±8.21 y Chronic stroke (>6mo since event), population with hemiplegia	I: Group watched a video (2min 30s) and then the physical training was applied for 12 min 30 s. 16 different tasks in total, difficulty of tasks adjusted depending on patient’s functional status and level. C: Group instructed to observe static landscape photos such as mountains, beaches, valleys, and countryside. Post videos, the same physical training program as the intervention group was performed. Adjunct: None specified.	15-min sessions, twice/d, 3 times/wk for 6 wk	Assessed at baseline, posttest 1 after 3 wk (before crossover), posttest 3 at 6 wk (after crossover) (i) TUG (ii) Dynamic Gait Index (iii) Weight distribution index (iv) Limit of stability	(i) NS (ii) NS (iii) NS (iv) SD between groups in favor of I group (*P*<.05). At posttest 1, between-group MD: 38.62 (95% CI, 17.33-59.91)
Lee et al^30^	Stroke	I: (AO+physical practice) n=12 Mean age ± SD: 62.8±7.4 y Mirror therapy group: n=11 Mean age ± SD: 57.27±5.7 y Only action observation training group: n=12 Mean age ± SD: 59.8±6.7 y Population with chronic stroke	I: Watched video (15min) and physical practice of the same actions for 15 min after. Mirror therapy group: Mirror therapy for 15 min/d and physical training of the same motions without a mirror for 15 min Action training group only: Action observation only for 30 min. This group watched a video of motions performed by others. No physical practice after. Adjunct: All groups received general PT twice/d for 30 min.	30 min 3/wk for 6 wk	Assessed at baseline and post intervention (i) Biodex Balance System: postural stability and falls risk were used to measure static and dynamic balance index (ii) Modified functional ambulation profile	Overall balance index significantly reduced in the I group (*P*<.05). (i) NS (ii) NS
Moon & Bae^32^	Stroke	I: n=7 (1F, 6M) Mean age ± SD: 59.1±10.0 y C; n=7 (4F, 3M) Mean age ± SD: 55.8±6.2 y Chronic (>12mo), population with hemiplegia, 1st stroke	I: Watched a backward walking video for 10 min, instructed not to imitate that actions while watching the video, rested for 10 min, then performed backward walking training for 20 min. C: Watched a landscape picture for 10 min, then performed backward walking training for 20 min. Adjunct: Both groups underwent conventional therapy for 30 min, 5 times/wk for 4 wk, consisting of functional, strengthening, and weight transfer exercises.	30 min 3/wk for 4 wk	Assessed at baseline and post intervention (i) Dynamic Gait Index (ii) 10MWT (iii) TUG	(i) SD between groups in favor of I group. Between-group MD: 2.00 (95% CI, 03.04-7.04) (ii) More significant improvement in I group. Between-group MD (m/s): 0.04 (95% CI, −0.17 to 0.25) (iii) SD in both groups, with more significant improvement in I group. Between-group MD: −3.04 (95% CI, −22.08 to 16.00)
Park et al^33^	Stroke	I: n=20 (10F, 10M) Mean age ± SD: 51.15±14.81 y C: n=20 (9F, 11M) Mean age ± SD: 48.65±12.81 y Chronic, population with hemiplegia	I: Watched videos (3min) of walking on a flat land, on a slope, and on steps. All executed by a healthy person and took a minute break afterward. Group then performed walking training for 5 min each of the same flat land, slope, and steps as in the video. Between each set of training they took 1-2–min break and in total the walking training took 20 min. C: Watched a video on nature. Had the same gait training as the other group for 20 min after. Adjunct: Prior to training the participants in each group received 30 min of general PT.	30 min/d 5 d/wk for 5 wk	Assessed at baseline and post intervention (i) Balance ability measure and training system (using biofeedback, AP1153 BioRescue, France): distribution of weight bearing on the paretic and nonparetic sides, the total distance of movements of the center point of the body and the area of the movements were measured (ii) TUG (iii) 10MWT	(i) SD between groups in favor of I group for limit of stability and sway speed. Between-group MD limit of stability (mm^2^): 2187.80 (95% CI, −142.03 to 4517.63); sway speed: −0.2(95% CI, −3.40 to −0.06) (ii) NSD (iii) NSD
Park et al^35^	Stroke	I: n=11 (3F, 8M) Mean age ± SD: 55.91±9.10 y C: n=10 (3F, 7M) Mean age ± SD: 54.80±12.22 y	I: Watched videos demonstrating 4 tasks for functional walking (10min). All aspects of walking tasks were demonstrated with 2 speeds (normal and 2 times lower) and presented from 3 angles. Watched video clips twice and executed the task for 20 min. C: Watched videos demonstrating landscape images (10min). Participants performed the walking tasks, which were the same walking tasks that participants in the I group practiced. Adjunct: All participants received functional training according to the daily routine schedule of the PT unit.	30 min 3/wk for 4 wk	Assessed at baseline and post intervention (i)10MWT (ii) Figure-of-8 Walk Test (iii) Dynamic Gait Index (iv) Gait symmetry score was measured using the GAITRite system	(i) SD between groups in favor of I group (*P*<.05). Between-group MD: −3.55 (95% CI, −33.84 to 26.74). (ii) SD between groups in favor of I group (*P*<.05). Median between I group difference: −3.50 (IQR, −12.60 to 2.00). Median between C group difference: −1.25 (IQR, −4.98 to 0.25) (iii) SD between groups in favor of I group (*P*<.05). Median difference in I group: 4.00 (IQR, 3.00-6.00); in C group 1.00 (IQR, −4.00 to 3.00) (iv) SD between groups in favor of I group (*P*<.05)
Park et al^34^	Stroke	I: n=12 (3F, 9M) Mean age ± SD: 57.33±6.89 y C: n=13 (6F, 7M) Mean age ± SD: 55.08±8.12 y Chronic (>6mo), population with hemiplegia, 1st stroke	I: Contents of videos consisted of a healthy male walking on even/uneven ground in a complex and unpredictable community environment. Each action presented from 3 different angles. Videos presented in 2 different filming speeds: normal and 50% normal. Video sound also provided. After the clips, the therapist asked the participants about the walking actions to ensure proper concentration. No physical practice after observation. C: Participants in the control group were asked to observe 4 different 30-min video clips of static landscapes. No physical practice after observation. Adjunct: All participants received functional training, which included walking training for 30 min, 5 times/wk for 4 wk.	30 min 3/wk for 4 wk	Assessed at baseline and post intervention (i) 10MWT (ii) Community walk test (iii) Activities-specific balance CIs (iv) Spatiotemporal parameters using the GAITRite analysis system using all the temporal and spatial parameters of gait to quantify the variance of gait	(i) SD between groups in favor of I group (*P*<.05). Between-group MD (m/s): 0.12(95% CI, 0.00-0.24) (ii) SD between groups in favor of I group (*P*<.01). Between-group MD (s): 741.34 (95% CI, 519.83-962.85) (iii) SD between groups in favor of I group (*P*<.01). Between-group MD: 5.53 (95% CI, 2.13-8.93). (iv) SD between groups in favor of I group (*P*<.05) in stride length, single support, and velocity
Sale et al^40^	Stroke	67 participants (26F, 41M) Mean age ± SD: 66.5±12.7 y I: n=33 C: n=34 Acute stroke (30d), moderate-severe upper limb paresis, 1st stroke	I: Viewed videos showing 20 different daily routine tasks carried out in the upper limb. Patients were presented with 1 task/d, starting from the easiest and ending in the most complex action throughout the 20 sessions. Each action consisted of 3 different motor sequences displayed in order of ascending difficulty and lasting 3 min each. After each sequence, the OT prompted the patient to perform the same movement over a time period of 2 min, providing help when needed. They received verbal instructions by the OT. The OT decided if physical assistance was needed. Both sessions were at least 60 min apart. C: Participants were shown 5 static images (without any animal or human being). A cognitive task was required to keep the patient’s attention at high concentration for a 3-min sequence; this was in the form of an unrelated image. Participants were then asked to perform the same limb movements to a standard sequence simulating those performed by the I group. OT provided physical assistance as needed. Both sessions were at least 60 min apart. Adjunct: All participants underwent inpatient rehabilitation consisting of at least 3 h/d of PT, occupational therapy, and speech and language therapy.	2×15-min daily sessions, 5 d/wk for 4 wk. Every missed session was retrieved.	Assessed at baseline (T0), post intervention (T1), and 4-5–mo follow-up (T2) (i) BBT (ii) Fugl-Meyer Assessment	(i) SD between groups in favor of I group at T1 (*P*=.012) and T2 (*P*=.031). Percentages of maximum recovery change at TI: I group 23%±21% (33) C group 11%±14% (34) Percentages of maximum recovery change at T2: I group 31%±22% (28) C group 19%±21% (31) (ii) SD between groups in favor of I group at T1 (*P*=.003) and T2 (*P*=.023). Percentages of maximum recovery change at TI: I group 40%±24% (28) C group 22%±25% (34) Percentages of maximum recovery change at T2: I group 56%±32% (28) C group 30%±51% (31)
Son & Kim^43^	Stroke	I: n=10 (6F, 4M) Mean age ± SD: 67.6±6.3 y C: n=10 (5F, 5M) Mean age ± SD: 66.7±6.8 y Chronic (at least 6mo), hemiplegia	I: Watched video clips of themselves performing balance and functional gait training (walking 3m or 10m, walking on an unstable supporting surface, and walking away from block and walking over block tasks) and performed 2 trials of physical training for 10 min each. Total duration of intervention was 30 min. C: See below. Adjunct: Both groups underwent general rehabilitation training.	30 min 5/wk for 4 wk	Assessed at baseline and post intervention (i) Muscle activity was evaluated with surface EMG; EMG electrodes were attached to the rectus femoris, bicep femoris, tibialis anterior, and gastrocnemius muscles (ii) TUG (iii) 10MWT	(i) SD between groups in favor of I group (*P*<.05) (ii) SD between groups in favor of I group (*P*<.05). Between-group MD: −1.10 (95% CI, −3.58 to 1.38) (iii) SD between groups in favor of I group (*P*<.05). Between-group MD: −1.90 (95% CI, −2.86 to −0.94)
Zhu et al^46^	Stroke	I: n=31 (13F, 18M) Mean age ± SD: 57.75±15.57 y C: n=30 (14F, 16M) Mean age ± SD: 56.89±14.93 y Subacute- chronic (<6mo), hemiplegia, 1st stroke	I: Watched a video showing a specific action of the upper limb and then perform the same action after. A total of 30 action videos were used. Each video was approximately 50 s in duration and depicted as seen straight on ( 20s), right above (15s), and right inside (15s). The repeated action was recorded 2-3 times at each angle. Each action video was numbered accorded to difficulty from 1-30. Videos of similar difficulty were grouped into 5 groups of 6 videos. They were instructed to try their best simulate the action with their affected limbs. C: See below. Adjunct: Patients in both groups received conventional drug treatment, PT, and OT for 205 h, 6 times/wk for a total of 8 wk.	30 min 6/week 8 weeks	Assessed at baseline and post intervention (i) Fugl-Meyer Assessment (ii) Barthel Index (iii) MAS	(i) SD between groups in favor of I group (*P*<.05). Between-group MD: 3.91 (95% CI, −1.58 to 9.40) (ii) SD between groups in favor of I group (*P*<.05). Between-group MD: 8.28 (95% CI, 1.12-15.44) (iii) SD between groups in favor of I group (*P*<.05). Between-group MD for elbow flexors: −0.60 (−1.02 to −0.18) Between-group MD (95% CI) for elbow extensors: −0.53 (95% CI, −1.00 to −0.06)
Zhu et al^47^	Stroke	I: n=16 (6F, 10M) Mean age ± SD: 57.75±16.75 y C: n=15 (7F, 8M) Mean age ± SD: 56.89±17.93 y Subacute (<3mo), unilateral limb hemiplegia	I: Watched a video showing a specific action of the upper limb and then perform the same action after. A total of 40 action videos were used. Each video was approximately 50 s in duration and depicted as seen straight on (20s), right above (15s), and right inside (15s). The repeated action was recorded 2-3 times at each angle. Each action video was numbered accorded to difficulty from 1-30. Videos of similar difficulty were grouped into 5 groups videos. Instructed to best simulate the action with their affected limbs. C: Traditional rehabilitation. Adjunct: Both groups received traditional rehabilitation training (exercise and occupational therapies).	30 min/day 6/week 8 weeks	Assessed at baseline, post intervention, and 2-mo follow-up (i) Fugl-Meyer Assessment (ii) Barthel Index (iii) Somatosensory evoked potential	(i) SD between groups in favor of I group (*P*<.05). Between-group MD: 5.79 (95% CI, 1.09-10.49) (ii) SD between groups in favor of I group (*P*<.05) Between group MD: 9.91 (95% CI, 1.57-17.65) (iii) Latencies of N9 and N20 of the hemiparetic side of patients in the VFT group were significantly shortened and the amplitudes were significantly improved (*P*<.05)

Abbreviations: AHA, Assisting Hand Assessment; AOT, action observation therapy; AROM, active range of motion; C, control group; DASI, dual-afferent sensory input; EMG, electromyograph; ETFES; electromyography-triggered functional electical stimulation; FES, functional electrical stimulation; GMFM-E, Gross Motor Function Measure-part E; F, female; I, intervention group (AOT); IQR, interquartile range; M, male; MS, multiple sclerosis; NFOGQ, New Freezing of Gait Questionnaire; NS, not significant; PD, Parkinson disease; OT, Occupational Therapist; PDQ-30, 30-item Parkinson Disease Questionnaire; PLP, phantom limb pain; PT, physical therapy; SF-MPQ, Short-Form McGill Pain Questionnaire; 6MWT, 6-minute walk test.

### Risk of bias assessment

The RoB assessment is summarized in fig 2. Twenty-two studies presented a low RoB in all domains. Six studies presented with an overall some concerns of bias: the bias arose from the randomization process in 5 studies, 17, 18, 19^,^^44^^,^^45^ while the bias in sixth study lay within the measurement of outcomes domain.^36^ Eight studies presented a high RoB in the measurement of outcomes domain.^21^^,^^24^^,^^26^, ^27^, ^28^^,^^30^^,^^43^^,^^47^ Two of the above studies had additional some concerns of bias in further domains,^26^^,^^30^ whilst a single study had a second high RoB in another domain.^28^ Because of the nature of the intervention, blinding of the treating therapists was not possible. Although this is an inherent source of bias, it is, however, unavoidable because of the direct therapist-patient interaction necessary for inpatient AOT.

### Outcome measures

A wide range of outcomes were reported throughout the studies and are considered under ICF framework.^11^ A total of 52 OMs are listed (table 2): activities and participation (n=31), body structure and function (n=24), and 2 evaluated environmental factors (n=2). Ten of the OMs assessed more than 1 domain.

**Table 2:** OMs and ICF domain

	Outcome Measure	Body Structure & Function	Activities+Participation	Environmental Factors	Personal Factors
Activities of Daily Living	Barthel Index		✔		
	Modified Barthel Index		✔		
Balance	Balance Index	✔			
	Berg Balance Scale		✔		
	Limit of stability	✔			
	Tinetti Scale		✔		
	Weight Distribution Index	✔			
Function (including muscle)	Fugl-Meyer Assessment	✔			
	FIM		✔		
	Grip strength	✔			
	Stroke Impact Scale	✔	✔		
	MAS	✔			
	Modified Parkinson Scale	✔	✔		
	Modified Tardieu Test	✔			
	Motricity Index	✔			
	Unified Parkinson Disease Rating Scale	✔	✔		
Joint health	Lequesne Index	✔	✔	✔	
	Osteoarthritis scales+pain	✔			
	Range of movement	✔			
	Western Ontario and McMaster Universities Index	✔	✔		
Mobility	6-min walk test		✔		
	10-m walk test		✔		
	Dynamic Gait Index		✔		
	Figure-of-8 Walk Test		✔		
	Freezing of gait episodes		✔		
	Freezing of Gait Questionnaire		✔		
	Functional ambulation capacity		✔		
	Gross Motor Function Measure-88		✔		
	Modified Functional Ambulation Profile		✔		
	New Freezing of Gait Questionnaire		✔		
	Timed Up and Go		✔		
	Walking ability questionnaire		✔		
Pain	Short-Form McGill Pain Questionnaire	✔			
	Visual Analog Scale	✔			
Quality of life	39-item Parkinson Disease Questionnaire	✔	✔	✔	
	Short-Form 36	✔	✔		
Upper extremity function	ABILHAND-Kids		✔		
	Action Research Arm Test		✔		
	Assisting Hand Assessment		✔		
	Box and Block Test		✔		
	Children’s Hand Experience Questionnaire		✔		
	Frenchay Arm Test		✔		
	Jebsen-Taylor Hand Function Test		✔		
	Melbourne Assessment Scale	✔	✔		
	Melbourne Assessment of Unilateral Upper Limb Function	✔	✔		
	Modified Ashworth Index	✔			
	Motricity Index	✔			
	Pediatric Reaching Test		✔		
	Tyneside Pegboard Test	✔	✔		
	Wolf Motor Function Test		✔		

Table 3 outlines the level of evidence of the OM within each condition. Eleven meta-analyses were possible on data for persons with orthopedic conditions, Parkinson disease, and stroke within the following OMs: Tinetti scale, Berg Balance Scale (BBS), Timed Up and Go (TUG) (in Parkinson disease and stroke), 39-item Parkinson Disease Questionnaire (PDQ-39), Box and Block Test (BBT), Fugl-Meyer Assessment, Modified Barthel Index, Wolf Motor Function Test, and 10-m walk test (10MWT).

**Table 3:** Levels of evidence for the OEs

Condition	Strong Evidence (Level 1)		Moderate Evidence (Level 2)		Limited Evidence (Level 3)		Conflicting Evidence (Level 4)
	Supported	Unsupported	Supported	Unsupported	Supported	Unsupported	
Amputee					McGill Pain Q (pain)		
					VAS (pain)		
Orthopedic			FIM (absolute functional efficiency score)		ROM (movement)	VAS (pain)	
		FIM (motor subscale)		WOMAC (pain subscale)	Barthel Index (functional status)		
		Tinetti Scale (gait and balance)		WOMAC (stiffness subscale)	Lequesne Index (severity of osteoarthritis- functional status)		
				WOMAC (function)	TUG (balance)		
				SF-36 (motor recovery)	SF-36 (mental health)		
Cerebral palsy			Gross Motor Function Measure-part E (walking, running, jumping)	MAS (spasticity)	Pediatric Reach Test (reach performance)	Modified Tardieu Scale	AHA (upper limb Function)
				Grip strength (Jamar dynamometer)		Ankle stiffness (electronic goniometer)	Melbourne Assessment Scale (upper limb function)
				ABILHAND-Kids (bimanual activities)			MUUL (upper limb function)
				Jebsen-Taylor Hand Function Test (manual dexterity)			
				Tyneside Pegboard Test (manual dexterity)			
Dementia						Neuro psychological tests (memory function and cognition)	
Multiple sclerosis			Handgrip strength (Jamar dynamometer)				
Parkinson disease	BBS (balance)		Tinetti part 2 (walking ability)	Tinetti part 1 (Balance)			TUG (functional mobility)
	FOG Questionnaire (FOG assessment)		6-min walk test (aerobic capacity and endurance)	Modified Parkinson Scale (mobility)			10MWT (walking ability)
	PDQ-39 (disease-specific health)			Spatiotemporal walking variables (walking ability)			
	UPDRS (motor and nonmotor abilities)						
Stroke	BBT (manual dexterity)		Wolf Motor Function Test (upper limb motor ability)	Frenchay Test (reaching ability)		Motricity Index (strength)	MAS (spasticity)
	Fugl-Meyer Assessment (upper limb function)		Figure-of-8 Test (walking skills)	Stroke Impact Scale (disability and quality of life)		ARAT (reaching ability)	
	Modified Barthel Index (activities of daily living)		6-min walk test (aerobic capacity and endurance)			Functional ambulation status	
	TUG (functional mobility)					Ambulation category	
	Dynamic Gait Index (balance and falls risk)						
	10MWT (walking ability)						

Abbreviation: ARAT, Action Research Arm Test.

### Musculoskeletal conditions

#### Amputees

One study with some concerns of bias evaluated the effect of AOT in the rehabilitation of bilateral amputees with phantom limb pain^44^ (see table 1).

(a) Body function and structure
  (i) Pain

There is level 3 evidence in favor of AOT in reducing phantom limb pain as evaluated by the McGill Questionnaire and visual analog scale (VAS) (see table 3). With respect to the information provided, it was possible to estimate the MD in both OMs. Significant between-group differences emerged for the McGill Questionnaire in favor of the AOT group, with scores decreasing more than the smallest detectable change of 5 points in this group only.^52^ Similarly, VAS score estimations revealed a between-group MD, with 73% of the AOT group demonstrating an MCID (≥20mm decrease) vs none in the mental visualization groups.

#### Orthopedic surgery

Three studies investigated the effect of AOT post total knee or hip replacements, 2 studies had a RoB with some concerns,^36^^,^^45^ and 1 study had a low RoB^15^ (see table 1).

(a) Body function and structure
  (i) Range of motion (ROM)
  (ii) Pain
  (iii) Stiffness

Level 3 evidence supports AOT in improving ROM, pain, and stiffness within the Western Ontario McMaster Universities Osteoarthritis Index (WOMAC) but does not support pain improvement scores in the VAS in patients with first-time hip and knee arthroplasty (see table 3). A single low-quality study reported a trend of greater ROM available in the AOT group, with large between-group posttreatment effect sizes reported (*d*>1.3), along with no between-group differences for pain in the VAS.^45^ A single lower-quality study reported a significant between group MD in favor of the AOT group in both of the subscales of the WOMAC (*P*<.001).^36^

(b) Combined activities and participation and environmental
  (i) Activities of daily living
  (ii) Physical function
  (iii) Walking ability
  (iv) Health status

Level 3 evidence does not support AOT as an effective intervention to improve functional status as assessed by the Barthel Index and Lequesne Index but does support motor recovery in the Short Form-36 Health Survey (SF-36) and the function scale of the WOMAC (see table 3). A single low-quality study found no between-group differences for the Barthel Index and Lequesne Index but did find a significant effect of time (*P*<.001) for motor recovery in the SF-36, with moderate between-group effect sizes at the end of treatment (*d*=0.76).^45^ A low-quality study,^36^ reported in participants with knee arthroplasty secondary to degenerative gonarthritis, significant between-group differences in the function scale of the WOMAC, again in favor of the AOT group with a between-group difference of −13.32, exceeding the MCID of 9.1 for the WOMAC function scale.^53^ Level 2 evidence supports functional improvements in the FIM as positive results are seen in a high-quality study, with FIM absolute functional efficiency score changes being significantly different, with a between-group MD of 6.4.^15^

**Fig 3:**
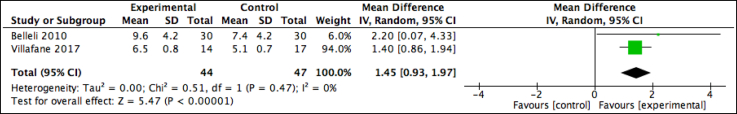
Pooled analysis for the Tinetti scale in patients with orthopedic conditions.

Level 2 quality evidence supports AOT in positively influencing gait and balance measures as evaluated by the Tinetti scale and FIM motor scores (see table 3). In the Tinetti Scale, a lower quality study found no between group differences,^45^ whilst a high-quality study found significant differences in changes in the Tinetti scale in favor of the AOT groups.^15^ A pooled analysis of these scores from a total of 91 patients revealed a significant positive effect size of 1.45 (95% CI, 0.93-1.97) in favor of the AOT group (fig 3), with a low heterogeneity (*I*^2^=0%), exceeding the MDC of 0.97 as referenced in the literature.^54^ Belleli et al^15^ also reported a significant change in the motor component of the FIM (*P*=.01) in the AOT group, with a clinically significant change in the absolute functional gain score (MCID>22),^55^ along with a reduction in the number of the walking aids needed (*P*=.01). Despite more patients in the AOT group being prescribed a walker at baseline, 96.7% were mobilizing with a single crutch at discharge vs the 73.3% in the control group (*P*=.01). Level 3 evidence is not in support of selecting AOT in improving balance or quality of life, as assessed by the TUG and SF-36, respectively (see table 3). A lower-quality study reported no significant between-group differences in the TUG,^36^ with both groups exceeding the MCID of 2.27 seconds.^56^ A separate lower-quality study reported no significant effect in the mental component of the SF-36.^45^

### Neurologic conditions

#### Cerebral palsy

Six studies examined the effect of AOT in improving upper limb function in the rehabilitation of children with cerebral palsy; 4 studies had a low RoB,^16^^,^^29^^,^^41^^,^^42^ 1 with some concerns of bias,^17^ and 1 with a high RoB^24^ (see table 1).

(a) Body structure and function
  (i) ROM
  (ii) Strength
  (iii) Spasticity and stiffness

Level 3 evidence does not support AOT in improving spasticity scores or ankle stiffness, as examined by the Modified Tardieu Scale and an electronic goniometer, respectively (see table 3). A single low-quality study demonstrated no significant between-group MD in children with diplegia in either measure.^24^ Level 2 evidence shows AOT to have no effect on spasticity in the Modified Ashworth Scale (MAS) or strength (see table 3). A single high-quality study found no significant between-group difference in MAS scores or grip strength assessed by the Jamar dynamometer in children with unilateral cerebral palsy.^42^

(b) Combined activities and participation and body function
  (i) Upper limb motor skills
  (ii) Unimanual and bimanual abilities
  (iii) Walking, running, and jumping

Level 4 evidence is found for the effectiveness of AOT in improving upper limb function as assessed by the Melbourne Assessment of Unilateral Upper Limb Function (MUUL), the Melbourne Assessment Scale, or the Assisting Hand Assessment (AHA) (see table 3). Three high-quality studies in children with unilateral cerebral palsy^29^^,^^41^^,^^42^ and a fourth low-quality study in children with hemiplegic or tetraplegic cerebral palsy^17^ evaluated the effect of AOT in improving AHA scores. Significant between-group changes in favor of AOT were reported in AHA scores in 2 of the studies.^17^^,^^41^ The changes in AHA scores exceeded the smallest detectable difference (>0.76 logits or 3.65 scores)^57^ in both studies, with Sgandurra et al^41^ reporting changes of 1.02 logits at the 6-month follow-up and Buccino et al^17^ recording changes of 5.73 in the AOT group at the 2-month follow-up. The other 2 studies did not demonstrate a significant between-group differences in children with unilateral cerebral palsy.^29^^,^^42^ The MUUL or Melbourne Assessment Scale was assessed in 1 low-quality^17^ and 3 high-quality studies.^16^^,^^29^^,^^41^ Significant between-group changes in MUUL scores were reported in 1 study^17^ but not in the second study^41^; the MD did not exceed the clinically significant threshold of 8.9% in either study.^58^ One study found that functional score gain in the Melbourne Assessment Scale was significantly different in favor of AOT, with an estimated 15-score difference,^16^ while the fourth study reported no between-group difference in the Melbourne Assessment Scale 2.^42^

Level 3 evidence supports AOT’s effectiveness in improving reach performance (see table 3). The mean values of the pediatric reaching test increased significantly more in the AOT group in a single low-quality study.^24^ Level 2 evidence does not support AOT in improving bimanual abilities improvements or manual dexterity in children with unilateral cerebral palsy as evaluated by the ABILHAND-Kids, Jebsen-Taylor Hand Function Test, and Tyneside Pegboard Test, respectively (see table 3). Two high-quality studies demonstrated no significant between-group difference in the ABILHAND-KIDS.^29^^,^^42^ A single high-quality study found no between-group differences for the Jebsen-Taylor Hand Function Test and the Tyneside Pegboard Test.^42^ Four studies which implemented a long-term follow-up found that the positive results seen post intervention continued in the long-term.^17^^,^^29^^,^^41^^,^^42^

Level 2 evidence supports the use of AOT in improving walking, running, and jumping activities as captured in significant between-group difference for the walking, running, and jumping abilities in the Gross Motor Function Measure part E.^42^

#### Dementia

One study with some concerns of bias examined the effects of observing hand function on cognition in older individuals with dementia^19^ (see table 1).

(a) Body structure and function
  (i) Neuropsychological tests

Level 3 evidence was not supportive of AOT for cognitive gains in populations with dementia (see table 3). No significant results were found in any of the memory function or cognition domains. Further analyses showed an improvement in face recognition tasks only.

#### Multiple sclerosis

A single study with a low RoB investigated the effects of AOT in adults with multiple sclerosis^39^ (see table 1).

(a) Body structure and function
  (i) Handgrip strength

Level 2 evidence supports the implementation of AOT in improving hand strength in persons with multiple sclerosis (see table 3). The right Jamar dynamometer score was significantly better in the AOT group vs the control group (*P*=.04), with only the AOT group exceeding the MCID value of 2.7 kg as reported for immune-mediated neuropathies.^59^

#### Parkinson disease

Five studies with a low RoB investigated the effect of AOT in patients with idiopathic Parkinson disease, stage 1-3 on the Hoehn and Yahr scale^12^^,^^23^^,^^31^^,^^37^^,^^38^ (see table 1). Four studies examined the effect of AOT on freezing of gait (FOG).^12^^,^^31^^,^^37^^,^^38^ The fifth study examined gait patterns, assessing spatiotemporal walking variables.^23^

(a) Activities and participation
  (i) Balance
  (ii) Walking ability

**Fig 4:**
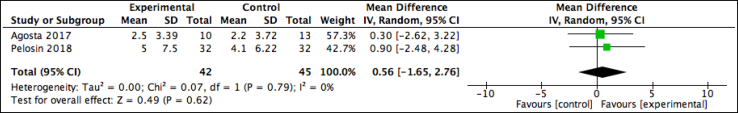
Pooled analysis for the Berg Balance Scale in patients with Parkinson disease.

Level 1 evidence supports the use of AOT in improving static and dynamic balance in patients with Parkinson disease (see table 3). The BBS and Tinetti part 2 were selected to assess balance. Three studies favored the AOT group in BBS scores at either short-term^31^ or long-term,^12^^,^^38^ revealing a significant effect for time (*P*<.001). A fourth study found no significant between-group difference in both the Tinetti and BBS.^37^ A meta-analysis of the BBS was only possible with 3 of the studies (fig 4), revealing a positive but nonsignificant effect size of 0.56 (95% CI, −1.65 to 2.76) in 89 participants and a low heterogeneity (*I*^2^=0%), with the MCID for this OM (1.9) falling within the limits of CIs.^60^

**Fig 5:**
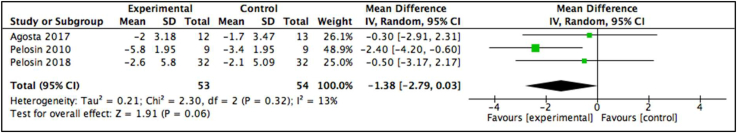
Pooled analysis for the FOG Questionnaire in patients with Parkinson disease.

Level 1 evidence supports the use of AOT in patients with Parkinson disease in improving FOG as evaluated by the FOG Questionnaire (see table 3). All 4 studies favored the AOT group,^12^^,^^31^^,^^37^^,^^38^ with significant between-group differences being reported immediately post intervention^12^^,^^31^ or in the long-term 4-week follow-up assessment.^37^^,^^38^ Additionally, Pelosin et al^37^ also found the number of FOG episodes in the AOT group to be significantly lower in the follow-up period 4 weeks post intervention (*P*<.001). A meta-analysis of the 3 studies pooling results from 107 participants revealed a low heterogeneity (*I*^2^=13%) and a significant positive effect size, with the intervention group decreasing in score by 1.38 times that of the control group (95% CI, −2.79 to 0.03) (fig 5).

**Fig 6:**
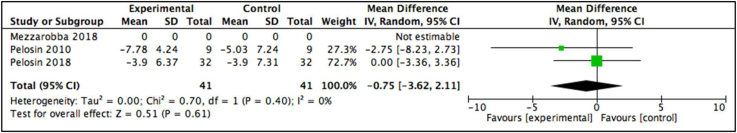
Pooled analysis for the TUG in patients with Parkinson disease.

Level 4 evidence is found for the use of AOT in improving functional gait and mobility as assessed by the TUG or 10MWT in 4 of the studies (see table 3).^12^^,^^31^^,^^37^^,^^38^ No between-group differences were found in the TUG in 2 studies,^31^^,^^37^ while Pelosin et al^38^ found the improvements to be maintained only in the AOT group at the 4-week follow-up. A meta-analysis was possible on the TUG scores in 2 studies; the pooled results from 82 participants revealed a nonsignificant effect of −0.75 (95% CI, −3.62 to 2.11) and a low heterogeneity (*I*^2^=0%)(fig 6). The lower value in the MCID range of 2-5 seconds falls within the CI range.^61^ Two studies found no between-group differences in the 10MWT,^37^^,^^38^ while 1 study^12^ found between-group improvements presented at an earlier time point in the AOT group, exceeding the MDC of 0.18m/s.^61^ Level 2 evidence supports the 6-minute walk test but does not support the Tinetti part 1 scale (see table 3) because 1 study found significant between-group differences in the 6-minute walk test at the second follow-up,^31^ while the second study, which had no physical practice of AOT, found no between-group difference in the Tinetti part 1.^37^

(b) Combined body structure and function, activities and participation, or environmental
  (i) Disease-specific health
  (ii) Functional abilities

**Fig 7:**
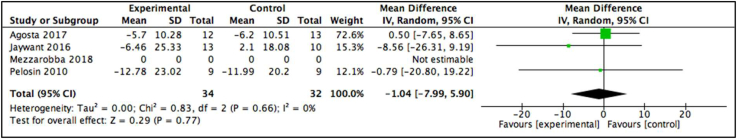
Pooled analysis for the 39-Item Parkinson Disease Questionnaire.

Level 1 evidence supports the PDQ-39, which assesses Parkinson disease–specific health, as indicated with favorable results in the AOT groups (see table 3). Three studies found significant improvements in the AOT group only, either in the short-term or at the 1- or 3-month follow-up.^12^^,^^23^^,^^31^ A fourth study, found no between-group differences.^37^ A meta-analysis for 3 of the 4 studies revealed low heterogeneity of the pooled studies (*I*^2^=0%) (fig 7). While results from the included 66 participants revealed a nonsignificant effect of −1.04 (95% CI, −7.99 to 5.90), the MCID (−4.72) for this OM does fall within the range of the CI.^62^

Level 1 evidence supports AOT in improving Unified Parkinson Disease Rating Scale (UPDRS) scores in individuals with Parkinson disease (see table 3). Two studies assessed motor and nonmotor abilities using the UPDRS.^12^^,^^31^ Performance improvements in the UPDRS II presented immediately post intervention in the AOT groups in both studies, with these being significant in the first and second follow-up (*P*<.05) in 1 study.^31^ Similarly, the positive findings in the UPDRS III were reported in both studies; one study reported a great effect size for AOT training over the control group,^31^ while the second study reported between-group MDs, with only the AOT group exceeding the MCID of −3.25 for this OM.^61^ These significant changes were maintained in the final follow-up assessment in both studies. Level 2 evidence does not support the modified Parkinson Assessment scale because a single study found no significant between-group difference^31^ (see table 3).

#### Stroke

Nineteen studies examined the effect of AOT within this population (see table 1). The effect of AOT was examined in terms of upper limb function (n=9), including 5 studies with a low RoB,^20^^,^^22^^,^^25^^,^^40^^,^^46^ 1 study with an unclear RoB,^18^ and 3 studies with a high RoB^21^^,^^26^^,^^47^; walking ability or balance (n=9), including 6 studies with a low RoB^13^^,^^14^^,^^32^, ^33^, ^34^, ^35^ and 3 studies with a high RoB^28^^,^^30^^,^^43^; or a combination of upper limb function and walking ability (n=1), including 1 study with a high RoB.^27^

#### Stroke: upper limb

(a) Body structure and function
  (i) Strength
  (ii) Spasticity

Level 3 evidence does not support AOT in improving upper limb strength as assessed by the Motricity Index, while level 4 evidence is found for the use of AOT in improving spasticity (see table 3). A single lower-quality study assessed strength via the Motricity Index and reported no between-group differences.^18^ Two high-quality studies measured spasticity using the MAS in patients with subacute first-time stroke.^25^^,^^46^ Conflicting results were found. The MD in 1 study showed no significant between-group difference (*P*>.05; 95% CI, −0.402 to 0.624),^25^ while the second study reported significantly better MAS scores post intervention in the experimental group vs the control (*P*<.05),^46^ exceeding the MCID of 0.76.^63^

(b) Activities and participation or combined body structure and function and activities and participation
  (i) Manual dexterity
  (ii) Upper Limb Function
  (iii) Activities of daily living

**Fig 8:**
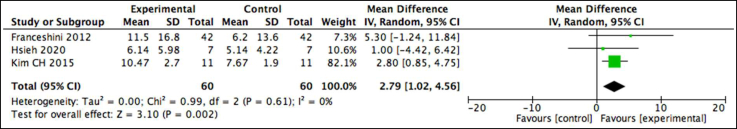
Pooled analysis for the BBT in patients with stroke.

Level 1 supports the use of AOT in positively influencing manual dexterity as assessed by the BBT. Four high-quality studies selected the BBT and reported significant changes in favor of the AOT group in populations with acute and subacute stroke.^20^^,^^22^^,^^25^^,^^40^ A meta-analysis was possible on 3 studies, pooling results from 120 participants, revealing a low heterogeneity (*I*^2^=0%) and positive significant effect of 2.79 (95% CI, 1.02-4.56) in favor of the experimental group (fig 8) but falling below the MCID of 5.5 blocks per minute.^64^

**Fig 9:**
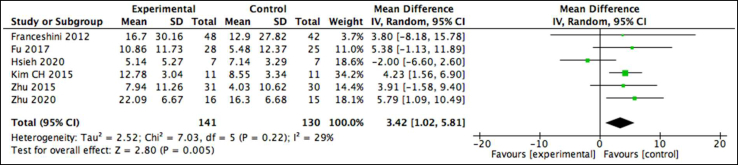
Pooled analysis for the Fugl-Meyer Assessment in patients with stroke.

Overall, level 1 evidence supports the use of AOT in improving upper limb function in patients with stroke. All 7 studies found positive improvements in the Fugl-Meyer Assessment in patients with subacute stroke, ranging from 30 days to 6 months post event. Significant between-group changes in favor of the AOT group were reported in 5 studies: 4 studies were high quality and 1 study was low quality.^21^^,^^25^^,^^40^^,^^46^^,^^47^ Two high-quality studies found no significant between-group differences.^20^^,^^22^ A meta-analysis was conducted on 6 studies. Unfortunately, because the results in 1 study were presented as percentages of maximum recovery potential, it was not possible to deduce an effect size for this study.^40^ The meta-analysis pooled results from a sample size of 271 participants and revealed both low heterogeneity (*I*^2^=0%) and a positive significant large effect size of 3.42 (95%, 1.02-5.81) in favor of the AOT group, (fig 9) with the MCID (5.2 points) falling within the CI margin.^65^

**Fig 10:**
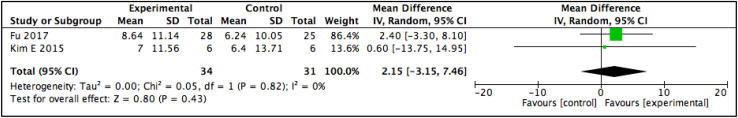
Pooled analysis for the Wolf Motor Function Test in patients with stroke.

Level 2 evidence supports the use of AOT in improving upper limb motor ability assessed by the Wolf Motor Function Test (see table 3). Two low-quality studies selected the Wolf Motor Function Test and reported significant between-group differences.^21^^,^^26^ The meta-analysis results from 65 participants revealed a nonsignificant effect size of 2.15 (95%, −3.15 to 7.46) and a low heterogeneity (*I*^2^=0%) (fig 10). The MCID of this OM (4.36), falls within the limits of the CI.^66^

Level 2 evidence does not support the use of AOT in improving reach test scores in the Frenchay Arm Test; and level 3 evidence does not support improvements in the Action Research Arm Test (see table 3). No between-group difference in individuals with an acute hemiplegic stroke was found for the Frenchay Arm Test in 1 high-quality study^20^ or the Action Research Arm Test in a separate low-quality study.^18^ Within the latter study,^18^ participants were recruited early after the stroke (3-31 days), and so the authors suggest perhaps the benefits from AOT are to be found in interventions introduced later on.

**Fig 11:**
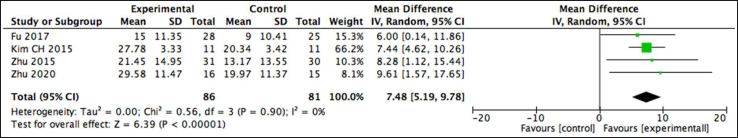
Pooled analysis for the Modified Barthel Index in patients with stroke.

Level 1 evidence supports the use of AOT in improving activities of daily living (ADL) in patients with subacute hemiplegic stroke (see table 3). Four studies, 2 high-quality^25^^,^^46^ and 2 of low-quality,^21^^,^^47^ used the Modified Barthel Index to assess ADL. After intervention, the changes in scores between the intervention and control groups were significantly different in all 4 studies (*P*<.05). The meta-analysis pooled results revealed a significant positive effect size of 7.48 (95% CI, 5.18-9.77) and a low level of heterogeneity (*I*^2^=0%) (fig 11), far exceeding the MCID of 1.85 for this OM.^67^

Level 2 evidence does not support AOT in improving disability and quality of life scores in patients with stroke (table 3). A single high-quality study selected the Stroke Impact Scale to investigate disability and quality of life, reporting no between-group difference.^22^

#### Stroke: walking ability and balance

(a) Activities and participation
  (i) Walking ability
  (ii) Balance

**Fig 12:**
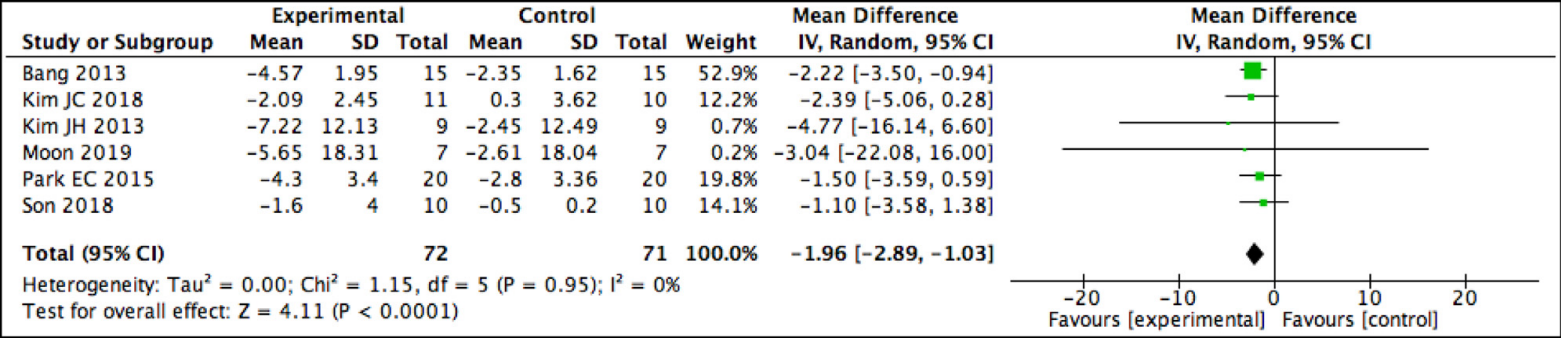
Pooled analysis for the TUG in patients with stroke.

Level 1 evidence supports the use of AOT in improving functional mobility and combined balance and falls risk, as assessed by the TUG and the Dynamic Gait Index, respectively. Four studies, 2 high-quality and 2 low-quality, which used the TUG, reported significant between-group differences (*P*<.05) in favor of the AOT group in chronic (>6 months) hemiplegic stoke.^27^^,^^33^^,^^34^^,^^43^ The fifth study,^28^ which was a crossover trial, reported TUG times significantly decreased in the AOT group between pretraining and post training 1 (*P*<.05) in chronic stroke (>6 months). While the sixth study reported a significant improvement in both groups, the AOT group demonstrated a more significant improvement in patients with chronic stroke (≥12 months).^32^ A meta-analysis revealed a significant effect size in the TUG, with the 72 experimental patients decreasing in scores by 1.96 seconds (95% CI, −2.89 to −1.03) greater than the 71 participants in the control group (fig 12). This score is below the MDC of 3.2 for this OM.^68^ Two high-quality studies reported significant between-group differences in favor of the experimental group in the Dynamic Gait Index,^32^^,^^35^ and a third low-quality study found no between-group difference.^28^ Only the score change in the intevention groups in both of these studies exceeded the MDC value (1.9) for this OM.^68^

**Fig 13:**
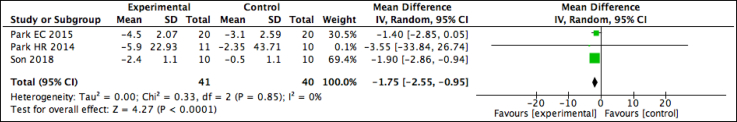
Pooled analysis for the 10MWT in patients with stroke.

Level 1 evidence supports the use of AOT in improving walking speed in individuals with chronic stroke (>6-12 months) as assessed by the 10MWT. Four of the 6 studies reported significant between-group differences in favor of the AOT group in the 10MWT; 3 studies were high-quality and 1 study was low-quality.^14^^,^^33^^,^^35^^,^^43^ The fifth^34^ and sixth studies,^32^ both high-quality, found significant improvements in both groups, with a significant between-group difference favoring the intervention group (*P*<.05). The meta-analysis possible within 3 of the studies using the 10MWT pooling 81 participants revealed low levels of heterogeneity (*I*^2^=0%). Overall, there was a significantly greater decrease in time in the experimental group, with a large effect of −1.75 (95% CI, −2.55 to −0.95) (fig 13), exceeding the MDC values of 0.1-0.2 depending on speed for this OM in patients with chronic stoke.^69^

Level 2 evidence supports the use of AOT in chronic stroke (>6-12 months) to improve motor planning in gait and walking distance as assessed via the Figure-of-8 Test and 6-minute walk test, respectively (see table 3). Authors reported significant between-group difference in 2 high-quality studies for the Figure-of-8 Test^35^ and the 6-minute walk test,^14^ with only the intervention group exceeding the MCID of 34.4m.^70^ Contrastingly, level 3 evidence does not support AOT in improving ambulation status because no signicant beween-group differences were found for the functional ambulation category^28^ or the modifed functional ambulation profile^30^ in 2 low-quality studies.

## Discussion

This systematic review included 1045 participants across 36 studies and examined the effect of AOT in rehabilitation of neurologic and musculoskeletal conditions. Level 1 and level 2 evidence supports the use of AOT in populations with orthopedic conditions, cerebral palsy, multiple sclerosis, Parkinson disease, and stroke. Level 1, representing strong evidence, supports of the use of AOT to improve OMs in Parkinson disease and stroke (see table 3). Within Parkinson disease, AOT therapy has been shown to result in improvements in balance scores, FOG, disease-specific health, and motor and nonmotor abilities. Similarly, consistently strong level 1 evidence demonstrated the effect of AOT in populations with subacute and chronic stroke in manual dexterity, upper limb function, balance, and walking ability. Level 2, representing moderate-quality evidence, advocates the implementation of AOT into rehabilitation to improve pain, stiffness, functional efficiency, gait, and balance in persons with orthopedic conditions and to improve grip strength in persons with multiple sclerosis. Moderate evidence shows walking, jumping, and running improvements in cerebral palsy, bimanual activities, dexterity, and spasticity in this population are not supported. Similarly, while AOT is supported for walking ability and aerobic capacity in Parkinson disease, improvements in spatiotemporal variables, mobility, and balance are not supported by moderate levels of evidence. Walking skills and aerobic capacity are also supported by moderate evidence in persons with stroke, as is upper limb motor ability, while reaching ability, quality of life, and disability go unsupported.

The OM used in the studies included cognitive, motor, and nonmotor assessments, including both functioning and disability components as outlined by the multidimensional ICF model. An excellent retention rate of improvements in the medium- to long-term was seen in 11 of the 12 studies that included a follow-up period, ranging from 1-6 months.^12^^,^^17^^,^^20^^,^^22^^,^^31^^,^^37^^,^^38^^,^^40^, ^41^, ^42^^,^^47^ This is a clear indication of the effect AOT has in promoting neuroplasticity and subsequent motor control improvements in rehabilitation.

Twenty-two studies presented with a low RoB, while the remaining 14 scored an uncertain or high RoB. Sufficient homogeneity of the studies allowed for 11 meta-analyses to be performed, the results of which revealed a significant effect in 7 of the OMs. Differing units in the reporting of OMs or insufficient information provided were the main limitations in performing further meta-analyses. While the meta-analyses of the BBS, TUG, and PDQ-39 in Parkinson disease and the Wolf Motor Function Test in stroke failed to show a significant effect, the MCID for these values did fall within the bounds of the CIs, thus illustrating that results can be statistically insignificant but clinically significant and so the clinician must not disregard the potential positive effect of treatment too hastily.^71^ Similarly, while the meta-analysis of BBT and TUG in stroke showed a significant effect of AOT, the effect size was below the reported MCID values for these OMs, again requiring judicious deliberation on the clinician’s behalf. Because effect sizes and sample sizes are interrelated, it is important to judiciously consider the sample sizes.

While not an aim of this review, strong psychometric properties are associated with the listed level 1 and level 2 OMs, further validating the positive results found within these measures. The BBS is the most widely and validated OM used to asses balance in populations with neurologic conditions and is associated with high reliability, validity, and responsiveness.^60^ The PDQ-39 is associated with good construct validity and meets the standard for acceptable reliability.^72^ The UPDRS and FOG Questionnaire are sensitive and reliable OMs for assessing treatment intervention.^73^ The BBT has excellent reliability in assessing hand function in individuals with stroke.^64^ Similarly, the interrater reliability of the Fugl-Meyer Assessment to assess motor recovery after stroke is high.^74^ The Barthel Index is a reliable, valid, and responsive OM to assess ADL in stroke.^75^ Excellent reliability is associated with the TUG and Dynamic Gait Index, with a significant correlation found between the 2 measures.^68^ Equally, the 10MWT is established as a reliable measure to assess walking speed in stroke.^73^ Both the WOMAC and the FIM are valid and reliable OMs in populations with orthopedic conditions.^76^^,^^77^

A wide range of AOT parameters were implemented across the studies, rendering it not possible to outline specific optimal parameters in the implementation of AOT. The length of sessions ranged from 10-60 minutes, the frequency varied from daily to twice a week, and the duration of studies spanned 8 days to 12 weeks. Within the 7 studies that demonstrated no positive changes in OMs in the AOT groups, 3 of these studies assessed children with cerebral palsy. It is evident that AOT is not supported within this population. Factors of consideration are the participants’ age and the length of the sessions. Age ranged from 3-10 years, with the mean age being 5 years. It has been postulated that the development of the MNS runs parallel with the motor experience of the observer^78^; it is possible that the combination of the reduced motor experience in children along with the reduced attention span may had led to the lack of progress with AOT.

Similarly, AOT was not supported in improving cognitive function in participants with dementia who observed hand movements.^19^ Hand movements stimulate cortical areas that are involved in sensorimotor and cognitive processes,^79^ but no physical practice post observation was incorporated into the study protocol, perhaps explaining the lack of any notable progress within the cognitive domains. A lack of activation of the MNS in individuals with Alzheimer disease, which accounts for the leading cause of dementia in older persons, has been found in functional magnetic resonance imaging studies.^80^ It is reasonable to suggest that if there is a lack of presence of the MNS, then therapies targeting this neuron system are unlikely to be beneficial. Interestingly, 1 study found cognitive functions in patients with multiple sclerosis to improve in the AOT group.^39^ Perhaps indicating AOT can have varying effects on cognitive function, depending on the underlying neurologic condition.

Motor imagery (MI) has been found to be effective in improving motor skills.^2^ The case for incorporating MI into AOT lies in the shared neural regions within the brain that activate during both forms of therapy.^81^ However, conflicting views exist regarding the benefit of incorporating MI in AOT programs. A single study directly compared the effect of combined MI and physical practice vs AOT and physical practice vs physical training alone.^27^ The authors reported that only the AOT group demonstrated significant improvements in OMs. A potential explanation for this is the fact that MI is dependent on an individual’s inherent capability for imagining movements.^4^ AOT, however, provides the clear motor representation of the task.

Individuals do not need to have an underlying neurologic or musculoskeletal disorder to benefit from AOT. Athletes and members of the general population have benefited from this form of therapy.^10^^,^^51^^,^^82^ While AOT has been widely applied in the field of neurologic rehabilitation, the question emerges why is it underexplored in musculoskeletal rehabilitation? We know that neurophysiological changes occur across the central and peripheral nervous systems in chronic musculoskeletal disorders, including sensorimotor cortical areas.^83^ Strategies known to optimize neuroplasticity in the rehabilitation of musculoskeletal conditions have been called for in the literature.^5^ Could AOT potentially offer the solution to the current inconsistency seen in the rehabilitation of chronic musculoskeletal conditions? The answer lies within further investigation of AOT in musculoskeletal conditions.

### Study limitations

The main limitations of this systematic review are the lack of large samples sizes, the medium to high RoB identified in a number of the studies, and the risk of selection bias because only English studies published within the last 12 years were included.

## Conclusions

AOT is suggested to be an effective tool in promoting neuroplasticity and motor learning, making it an important and valid consideration for the clinician. The benefit of incorporating AOT training into rehabilitation programs where motor and nonmotor improvements are a desired outcome is strongly supported in populations with Parkinson disease and stroke and moderately supported in populations with orthopedic conditions and multiple sclerosis. AOT has been considerably less explored in musculoskeletal conditions. No conclusions can be drawn regarding optimal parameters of implementation for AOT.
